# Development and Validation of an ISA100.11a Simulation Model for Accurate Industrial WSN Planning and Deployment

**DOI:** 10.3390/s21113600

**Published:** 2021-05-21

**Authors:** Zoltan Padrah, Andra Pastrav, Tudor Palade, Ovidiu Ratiu, Emanuel Puschita

**Affiliations:** 1 Control Data Systems S.R.L., Liberty Technology Park, 21 Garii Street, 400267 Cluj-Napoca, Romania; Zoltan.Padrah@cds.ro (Z.P.); Ovidiu.Ratiu@cds.ro (O.R.); 2 Communications Department, Technical University of Cluj-Napoca, 28 Memorandumului Street, 400267 Cluj-Napoca, Romania; Andra.Pastrav@com.utcluj.ro (A.P.); Tudor.Palade@com.utcluj.ro (T.P.)

**Keywords:** industrial WSN, ISA100.11a model, ns-3, WSN

## Abstract

During the planning, design, and optimization of an industrial wireless sensor network (IWSN), the proposed solutions need to be validated and evaluated. To reduce the time and expenses, highly accurate simulators can be used for these tasks. This paper presents the development and experimental validation of an ISA100.11a simulation model for industrial wireless sensor networks (IWSN). To achieve high simulation accuracy, the ISA100.11a software stack running on two types of certified devices (i.e., an all-in-one gateway and a field device) is integrated with the ns-3 simulator. The behavior of IWSNs is analyzed in four different types of test scenarios: (1) through simulation using the proposed ISA100.11a simulation model, (2) on an experimental testbed using ISA100.11a certified devices, (3) in a Gateway-in-the-loop Hardware-in-the-loop (HIL) scenario, and (4) in a Node-in-the-loop HIL scenario. Moreover, the scalability of the proposed simulation model is evaluated. Several metrics related to the timing of events and communication statistics are used to evaluate the behavior and performance of the tested IWSNs. The results analysis demonstrates the potential of the proposed model to accurately predict IWSN behavior.

## 1. Introduction

Wireless sensor networks (WSNs) have become increasingly popular in the industrial domain as they collect and transmit data about the environment and provide a series of advantages that make them easier to expand than the wired networks: configuration flexibility, support for mobility, and reduced infrastructure weight [1].

This paper focuses on a particular category of WSNs, namely the industrial ones. Industrial WSNs (IWSNs) are addressed by several standards, including ISA100.11a [2] (IEC-62734 [3]), WirelessHART (IEC-62591) [4], ZigBee [5], Bluetooth Mesh Networking [6], Wireless Networks for Industrial Automation-Factory Automation (WIA-FA) (IEC-62948) [7,8], and Process Automation (WIA-PA) (IEC-62601) [9].

**Problem statement.** IWSNs are installed in environments having strict security, control, and safety measures. Such IWSNs are typically located on chemical plants, refineries, and maritime platforms, and their deployment is an iterative process including several steps that need to be carefully prepared and precisely executed. As such, during the deployment lifecycle of an IWSN, which includes defining objectives, factory survey, selecting candidates, designing a solution, and deploying and monitoring the network, a detailed analysis of the IWSN needs to be performed [1,10]. Such analyses become time-consuming and expensive when they rely on acquiring and managing sets of physical equipment and performing tests in specific locations. To overcome this issue, some of the tasks can be performed using highly accurate simulators and/or Hardware-in-the-loop (HIL) testbeds. In system planning activities, simulators are used to validate the candidate solution. Field device selection and network integration can also be validated using simulators and/or HIL testbeds. Moreover, realistic simulations are useful in predicting and evaluating the operational performance of IWSNs without deploying physical devices in testing scenarios. Therefore, simulations represent a fast and cost-effective way to determine the behavior of the network beforehand and are highly recommended for IWSN planning, design, deployment, and monitoring [1].

Ideally, simulations should be so realistic and accurate that a simulated IWSN and the physically deployed replica should behave identically under all circumstances arising during the lifetime of the IWSN.

**Scope of the paper.** In this context, this paper aims to propose and evaluate an enhanced IWSN simulation model that integrates the ISA100.11a communication stack functionalities with the ns-3 network simulator [11].

**Methodology.** The following steps were carried out to integrate the accurate ISA100.11a simulation model with ns-3:

- Porting a proprietary ISA100.11a software stack that runs on certified commercial devices to the same operating system as the one running the ns-3 simulator;
- Defining the interfaces between the ported ISA100.11a software stack and the ns-3 environment;
- Integrating the ported ISA100.11a software stack with the ns-3 simulation environment;
- Functional testing of the ISA100.11a simulation model for IWSN network formation and data collection.
- Iterative refinement of the ISA100.11a integrated simulation model for accurate modeling of a physical operational IWSN using HIL scenarios.

**Test and validation.** The proposed ISA100.11a simulation model is used to implement a basic IWSN with an infrastructure device and a field device. To validate the accuracy of the implemented model, we compare the behavior and performance of the simulated network with those obtained in the following hardware-based setups that replicate the same network:

- Physical network—in this scenario, the infrastructure device and the field device are real hardware devices, i.e., CDS VR950 gateway and CDS VS210 node.
- Gateway-in-the-loop—in this scenario, the physical infrastructure device is used to manage the simulated ISA100.11a network.
- Node-in-the-loop—in this scenario, the simulated infrastructure device is used to manage a physical ISA100.11a network.

Moreover, the scalability of the simulation model is tested by means of 5-node and 10-node networks both physically deployed and simulated. By scalability of the simulator, we mean its capability to correctly simulate the IWSN behavior as the number of the ISA100.11a devices is increased. In each scenario, the network performance is evaluated in terms of specific network formation and operation metrics.

**Originality and contributions.** The originality of this work consists of the highly accurate integration of the ISA100.11a software communication stack with ns-3. As such, in a unique approach, the same software stack found on the ISA100.11a compliant hardware devices is used in both the simulated scenarios and the experimental testbed replicas.

This paper is an expanded version of the conference paper [12] presented at the 2020 International Workshop on Antenna Technology (iWAT 2020). This work has been significantly extended, the major contributions being as follows:

- Contribution 1: A more thorough state-of-art is included, presenting the available IWSN simulation solutions and HIL approaches, highlighting the need for a reliable simulation tool.
- Contribution 2: An enhanced set of metrics is used to evaluate the performance of the proposed simulation model, in comparison with the hardware-based scenarios.
- Contribution 3: An extended set of experiments to test the functionalities of the gateway and node is performed by means of the two HIL scenarios (i.e., Gateway-in-the-loop and Node-in-the-loop). The HIL scenarios show that the ISA100.11a devices implemented by the simulator are the equivalents of the physical devices. In addition to showing that all the functionalities of physical devices have been successfully integrated with the simulator, the metrics collected from the HIL scenarios allow us to quantify the similarity in performance between the physical and simulated devices.
- Contribution 4: An extended set of experiments including real infrastructure and up to ten real field devices validating the accuracy and scalability of the proposed IWSN simulated model.

The rest of the paper is organized as follows. Section 2 indicates the key aspects of a realistic IWSN model. Section 3 presents existing IWSN simulation tools and indicates the main requirements of a reliable simulation environment. Section 4 briefly presents the ISA100.11a standard. Section 5 describes the implementation of the proposed ISA100.11a model and its integration with ns-3. Section 6 presents the test scenarios, while Section 7 highlights the performance evaluation results. Finally, Section 8 concludes the paper, emphasizing the potential of the implemented ISA100.11a model.

## 2. Industrial WSN Accurate Modeling

An accurate/realistic IWSN model is an IWSN model with externally observable behavior matching the behavior of a physical IWSN. This accuracy translates into an identical start-up sequence, communication characteristics, and response to changes in the physical topology or radio propagation conditions. Specifically, we consider the following aspects of a realistic IWSN modeling:

- The external interface and used communication protocol are the same for both the simulated and for the physical IWSN. This includes the case where an unmodified external control system receiving data from a physical IWSN can use a simulated IWSN as a source of the same data, with minimal configuration changes (e.g., changing the IWSN’s address in the control system’s configuration). From the control system’s point of view, the physical and simulated IWSN are the same.
- The start-up sequence and timing (synchronization) for a simulated and physical IWSN are similar. This includes the moments when devices become operational and when the first collected data samples arrive at the external interface of the IWSN.
- The QoS evaluation parameters (e.g., data throughput, transmission delays, packet size of the collected and transmitted data) are the same for both simulated and physical IWSNs.
- The provisioning data used for configuring a simulated IWSN can be trivially applied in the deployment of a physical IWSN.
- For a given set of field device positions, the network topology of a simulated IWSN is identical to that of a physical IWSN.
- After deployment, the physical and simulated IWSNs can be configured at run time by means of the external IWSN interface. The latency and bandwidth of message exchanges in this situation is identical between simulated and physical IWSN. For example, such additional configuration might be performed by the external control system for fine-tuning the data transmission period of a specific field device.
- In case there are changes in the radio propagation conditions or in the physical topology of the IWSN, the simulated and the physical IWSN react identically.

Through quick and efficient modeling, a realistic IWSN simulator helps solving various problems in different stages of the IWSN’s development, as follows.

In the planning phase, the IWSN simulator can be used to select the physical locations where field devices should be placed and to evaluate the feasibility of different network topologies. Moreover, it helps estimating the communication bandwidth and latency of a given candidate solution.

In the implementation phase, the provisioning data can be created inside the realistic simulator and exported to the field devices to be deployed. The start-up sequence and the integration of the IWSN with external systems can be implemented and validated using the realistic simulator.

In the deployment phase, for fine-tuning an IWSN, configuration changes can be validated in a realistic IWSN simulator. In addition, in case of changes in propagation conditions, the realistic simulator can predict the effects on the IWSN performance.

## 3. Review of IWSN Simulation Tools and Validation Methods

This section provides a concise and precise description of the experimental results, their interpretation, as well as the experimental conclusions that can be drawn.

In terms of accuracy, the widely used WSN and IWSN simulators [13] can be divided into two categories:

- Discrete-event network simulators that rely on generating events at certain moments of time: events that can, in turn, generate additional ones during the simulation. As such, a trade-off between computational efficiency and simulation accuracy is achieved. Such a simulator is OMNeT++ [14] together with specific frameworks usable with it such as Castalia [15], INET framework [16], MiXiM [17], InetManet [18], Riverbed Modeler (former OPNET Modeler) [19], QualNet simulator [20], TOSSIM [21], and ns-3 [11]).
- Cycle-accurate simulators that simulate each CPU cycle of a wireless device. These simulators require more computational power but are highly accurate, and they allow for the same binary files to run in the simulator and on real devices. Such simulators are Avrora [22] and Cooja [23].

### 3.1. Latest IWSN Simulation Solutions

The simulators listed above have been used to carry out several studies in the domain of IWSNs. We summarize here the most relevant works, emphasizing the accuracy of the simulations. We analyze the level of detail implemented in the simulations (as a greater level of detail allows more accurate simulations) and the metrics used during the simulator evaluation.

The work in [24] presents a WirelessHART implementation in OMNET++ for experiments concerning security. The model implements the full protocol stack and the Network Manager, using InetManet [18] features for the physical layer. This shows that implementing a full IWSN protocol stack in a simulator is feasible.

In [25], the authors modify a model of an industrial process (the widely used Tennessee Eastman Process Control Challenge Problem [26]) by applying wireless communication links in the model between sensors, actuators, and the process controller. Simulation results show that the process can operate by using wireless communication links. The effect of various wireless configurations on the operation of the process is evaluated. The used simulator is OMNET++ and models from INET and MiXiM frameworks are used for address resolution protocol (ARP) and wireless channel template, respectively. The simulation employs the WirelessHART protocol, and it focuses on the Physical (PHY) and Media Access Control (MAC) layers. The archived version of the source code of simulations is available online at [27]. The evaluated performance metrics are the deviation of process variables from their nominal values and the expected time for which the plant can operate normally. The packet error rate on the RF communications and the placement of the Access Points have been modified during simulations. This simulator does not implement a complete WirelessHART protocol stack nor the functionalities of a WirelessHART gateway.

In [28], Castalia and Pymote are used for simulating ISA100.11a, WirelessHART, and ZigBee networks. The metrics used for performance evaluations are throughput, number of packets transmitted, lost, and received, and device energy consumption. During simulations, the readily available generic models have been used (i.e., the IEEE 802.15.4 MAC and PHY layer models for ISA100.11a simulations and a generic “Throughput Test” application). Not all layers of the communication stack have been implemented, nor the functionalities of an ISA100.11a or WirelessHART gateway.

The work in [29] presents an ISA100.11a model for ns-3, implementing the physical and data link layers of the standard and a simplified application layer. The corresponding source code is available online at [30].

Avrora [22] is a cycle-accurate simulator for WSNs. It can handle as many as 25 nodes in real time. It is targeted for high timing accuracy with increased performance compared to ATEMU [31]. It achieves this by extracting fine-grained parallelism inside the simulation of a WSN.

A mathematical model for energy consumption of wireless sensor nodes is presented in [32]. It considers the energy consumption of communications, acquisition, and processing.

The importance of simulating the effects of CPU load on the IWSN is assessed in [33]. The ns-2 simulator [34] is integrated with the RTSim [35] software to accurately simulate wireless sensor nodes. The IEEE 802.15.4 standard is used for evaluation.

A discussion on the deployment of ISA100.11a and WirelessHART IWSNs in a refinery is presented in [36]. For assisting the deployment, a new simulator called RF Propagation Simulator (RFSim) has been created and used. It predicts the quality of the RF signal on the premises of the industrial installation. The simulation results are compared with onsite measurements and look promising. The authors note that most of the published work on sensor network deployment limits itself to 1D or 2D environment, and 3D environments are considered an open issue.

A complete WirelessHART stack and gateway implementation for the ns-2 simulator [34] is presented in [37]. To validate the model, the authors set up similar networks with real hardware and within the simulator. The performances of the two networks (i.e., simulated and real) are evaluated in terms of reliability in the network (i.e., failed transmission ratio and average of received signal level), communication scheduling and network throughput, real-time data transmission (i.e., end-to-end delay and interval between consecutively received packets), and energy consumption in the network. Management efficiency is evaluated in terms of overhead and delay for performance during joining and service request procedures. The simulator performance in multi-hop mesh networks is also assessed, highlighting the response of the network in case of node and link failure. Moreover, the data delivery ratio is evaluated for three lossy networks. The experiments showed that the simulated results are similar to the results obtained in real networks and that the proposed model is versatile and usable in diverse scenarios.

To enhance the simulation results, hardware-related aspects can be integrated into the simulation scenarios. This concept is known as Hardware-in-the-loop.

The TOSSIM simulator is integrated with physical wireless nodes at the radio communication level in [38]. The work demonstrates the feasibility of a WSN consisting of both simulated and physical nodes. This is achieved by using a pair of physical Dual Base Stations as a bridging device between the simulated and physical environments. The simulation runs in real time. The approach allows unmodified nodes to communicate with purely simulated nodes, but the number and location of the physical nodes are being constrained by the number and capabilities of bridging devices.

In [39,40], the authors use the Software-in-loop simulation technique to evaluate the software to be deployed in a WSN and to facilitate the deployment of a WSN. This is implemented by integrating a simulated environment and sensor device with the software that will run on deployed wireless sensor nodes.

The work in [41] presents how radio hardware can be integrated into a simulation. In this Radio-in-the-Loop (RIL) approach, the IEEE 802.15.4 Physical Layer and the Physical Medium are implemented by real hardware and physical phenomena, respectively. The communication layers above these, including the IEEE 802.15.4 MAC layer, are implemented by software. As such, the results of the experiments are more realistic than the results of pure simulations. The authors employ the OMNeT++ network simulator, and the interface toward the hardware is based on Linux/Unix inter-process communication and the PcapNG data format. The radio used is the IEEE 802.15.4 compliant ATmega128RFA1, running a special application on Contiki OS [42]. However, this work does not measure the latency introduced by the adaptation between the software and hardware parts of the simulation. For some IWSN protocols (e.g., ISA100.11a), the latency at the MAC layer is critical for the proper functioning of the slotted transmission scheme; excessive delays could make the communication impossible.

### 3.2. Limitations of Current IWSN Simulation Solutions

In our opinion, an IWSN simulator must be accurate (to correctly predict the behavior of the physically deployed IWSN system), fast (and therefore computationally efficient), and versatile (to accommodate different types of use-case scenarios). To this extent, the existing network simulators are not comprehensive, as they provide simplified models that lack accuracy or focus only on certain features of the network, leading to discrepancies between the behaviors of the simulated and physically deployed networks. Moreover, some models are suitable only for simulations, which makes them difficult to use in HIL scenarios. Table 1 synthesizes the main features of the existing IWSN simulation solutions in comparison with the model proposed in this paper.

As a result of the simplifications, the use of the existing models could lead to false predictions. In contrast, by integrating a field-tested communication stack (i.e., according to certified commercial devices) into the simulator, it should be possible to generate more reliable results. Having a realistic communication stack available in the simulation tools allows an earlier start of the wireless systems integration, by using the simulator interfaces toward external systems. In addition, an accurate simulation tool should help the industry by providing aid in the deployment and maintenance of IWSNs.

In the domain of generic WSNs, there are some cycle-accurate simulators (such as [21,22,23]) that simulate a software stack very similar to the software running on real devices, but these simulators do not implement standardized industrial communication protocols such as ISA100.11a or WirelessHART. To the best of our knowledge, the work in [37] presents the most thorough IWSN simulation model so far, but it is bound to the WirelessHART standard and does not consider a HIL validation method. NIVIS LLC [43] released an ISA100.11a open-source implementation but is not compatible with a simulator and runs on hardware that is not commercially available. An ISA100.11a model for ns-3 is available in [30] but cannot run on real hardware.

In this paper, we propose a comprehensive IWSN simulation model integrated with ns-3 that implements the ISA100.11a communication stack available on certified commercial devices. We make use of HIL scenarios to validate the implementation accuracy of the IWSN entities (i.e., ISA100.11a field and infrastructure devices).

To validate the overall IWSN model implementation, we compare the simulated IWSN behavior to that of the physical network replica.

### 3.3. ns-3 Simulator Selection Criteria

In our opinion, to facilitate the integration of a simulation model, the simulation environment must be open-source, actively maintained, mainstream, and general purpose. Moreover, to achieve high model accuracy, the environment should allow the integration of the same software stack running on real devices. As such, it should be compatible with C or C++ (which is used to implement the software running on the microprocessor of the ISA100.11a devices) and able to integrate proprietary C and C++ source code into the simulation.

Table 2 synthesizes the relevant criteria for selecting the simulation environment.

Given the above-mentioned requirements, we consider ns-3 [11] to be the best candidate for integration with the ISA100.11a IWSN simulation model. It is an open-source, actively maintained general-purpose simulator that integrates natively with C, C++ source code.

## 4. ISA100.11a Wireless Communication Standard

### 4.1. ISA100.11a Devices

An IWSN defined by the ISA100.11a standard consists of two categories of devices: field devices and infrastructure devices. Each device can implement one or more roles specific to their category.

Field devices can implement the Input/Output role, denoting the capability of collecting sensor data or managing actuators and the router role, denoting that they are capable of forwarding traffic to other field devices.

Infrastructure devices can implement System Manager, Security Manager, Gateway, and Backbone Router roles. The System Manager is responsible for all the communication-related aspects of the network, including the configurations necessary for devices to join the network, the set-up of the radio network, the management of Quality of Service (QoS) requirements of devices, and ensuring redundancy inside the network. The Security Manager handles the security-related functions of the network, including the authentication of devices and management of cryptographic keys. The Gateway provides standardized methods to get data out of the IWSN and to externally access the devices inside the IWSN, typically in order to configure non-communication-related aspects of devices—for example, the sampling rate of the data collected by devices. The Backbone Router provides means of communication between the wireless network and the Infrastructure Devices.

**Figure 1:**
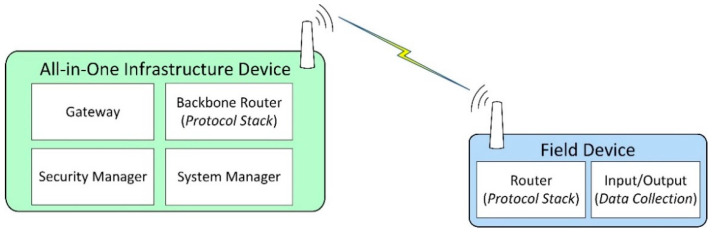
Roles and associated functionalities of ISA100.11a devices.

Figure 1 depicts a simplified ISA100.11a IWSN, showing the roles and functionalities associated with each device.

The IWSN is formed by an all-in-one infrastructure device, which in this case implements the ISA100.11a roles of System Manager, Security Manager, Gateway, and Backbone Router. An all-in-one infrastructure device might include functionalities not required by the ISA100.11a standard, for example, external protocol adapters.

Most field devices implement data collection functionalities. Data collection means that the devices include one or more transducers, and at some moments in time, the value is read from the transducer(s) and is inserted in the ISA100.11a protocol stack for transmission toward the infrastructure device. This data collection mechanism is typically integrated into the application layer of ISA100.11a by the means of User Application Processes (UAPs) and inside the UAPs by specialized process industries user application objects such as Analog Input Objects, Analog Output Objects, Binary Input Objects, and Binary Output Objects. The data from such objects are transmitted to the infrastructure device, where it reaches the Gateway module. External automation systems connect to the Gateway module through the External Protocol Adapter and retrieve the data.

### 4.2. Communication Stack

Table 3 summarizes the protocol layers defined in the ISA100.11a standard in the context of the OSI reference model.

Each device in an ISA100.11a IWSN implements the protocol layers defined by the ISA100.11a standard. Field devices and Backbone Routers implement all the protocol layers while the System Manager, Security Manager, and Gateway do not implement the physical and data link layers. The PHY layer and MAC sub-layer implement the IEEE 802.15.4 standard [49] specifications.

On top of the MAC sub-layer, the ISA100.11a standard defines a MAC extension sub-layer and an upper data link layer. These two layers use some of the functionalities defined by IEEE 802.15.4 (e.g., frame transmission and reception) and add extra functionalities (e.g., periodic transmission and reception opportunities by introducing ISA100.11a super-frames and links, routing inside an ISA100.11a IWSN using source routing and graph routing, authentication of the received frame using AES cryptography, or performing time synchronization between wireless devices during acknowledged (ACK) data transmissions).

The network layer of ISA100.11a performs addressing and routing. It uses the IPv6 addressing scheme. To differentiate between the QoS classes, the network layer is aware of the ISA100.11a contracts and passes this information to the data link layer to enable traffic prioritization.

The transport layer multiplexes traffic between the network layer and multiple Transport Layer Service Access Points (TSAPs), and it is capable of handling the encryption, decryption, and authentication of traffic passing through it. With each TSAP, an Application Sub-Layer (ASL) entity exchanges protocol data units (PDUs) with the application layer. The ASL sub-layer provides access primitives to the object-oriented structure of User Application Processes (UAPs). These primitives include read attribute, write attribute, execute method, publish request, and publish notify. Each ASL entity is connected to one UAP. On each ISA100.11a device, at least one UAP must exist: the Device Management Application Process (DMAP). The DMAP exposes the configuration interface of the device in order to be integrated into the network. Typically, sensor or actuator devices have an additional UAP exposing the sensing or actuating capabilities of the device to the ISA100.11a network.

## 5. ISA100.11A Communication Stack Implementation in ns-3

Ns-3 is a discrete event-based simulator, written in C++, but simulations can also be defined in Python. It uses WAF [50] as a build system and offers models for various networking protocols and applications, including wireless technologies. Conceptually, a simulation scenario consists of several Nodes, which run Applications and communicate through NetDevices, which are attached to Channels. During simulations, various statistics, metrics, and raw communication data can be collected, and various simulation events can be logged. The collected data can be further analyzed with external tools.

### 5.1. Implementation of an ISA100.11a IWSN

In this work, we integrate a proprietary ISA100.11a software stack developed by the Control Data System (CDS) [51,52,53] with ns-3. This software stack runs on certified commercial devices and has been tested for interoperability with devices from different vendors, such as Yokogawa, Honeywell, or Draeger.

**Figure 2:**
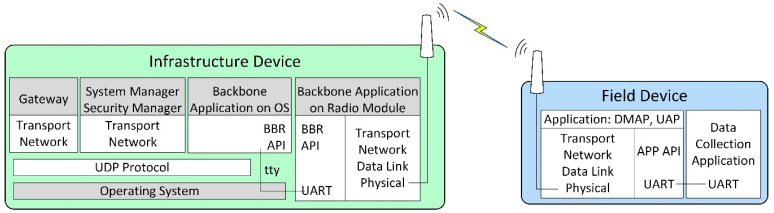
The internal architecture of physical ISA100 IWSN devices.

The internal architecture of physical ISA100.11a IWSN CDS devices employed by our hardware-based scenarios is illustrated in Figure 2.

The implementation architecture is based on the CDS VR950 gateway [52] as the infrastructure device and the CDS VS210 development board [53] as the field device. These devices are commercially available, and they contain an ISA100.11a certified radio module with a software stack.

The VS210 development board contains two parts: (1) the radio modem running the ISA100.11a stack, including the application layer with DMAP and UAP, and an integration API denoted APP API, which is exposed through a Universal Asynchronous Receiver/Transmitter (UART) interface; (2) the data collection application, running on a microcontroller separate from the radio modem and communicating with the radio modem through a UART interface.

The VR950 gateway also consists of two major parts:

- The transceiver of the Backbone Router, which is connected to the antenna of the gateway. It runs a complete ISA100.11a protocol stack, and it includes an integration API with the rest of the gateway, which is denoted here as BBR API. Its application layer only has a DMAP. The communication with the other part of the gateway is performed through a UART.
- The operating system environment inside the Gateway, which is running on an embedded computer. The operating system is based on Linux. This part is connected to the transceiver over a UART (tty) interface, and it runs several network management applications that implement the gateway logical modules. These applications are the Gateway application, the System Manager (SM) and Security Manager application, and the Backbone Application on OS (BBR). All of these applications communicate with each other through User Datagram Protocol (UDP). The Gateway and SM applications include the Network, Transport, and Application layers defined by ISA100.11a, while the BBR application performs the translation between the UART and UDP communication. The gateway has IP connectivity through an Ethernet interface.

This work considers the ISA100.11a software stack implementations of the field device and the all-in-one infrastructure device.

### 5.2. The ISA100.11a IWSN Simulation Model

The goal of the proposed simulation model is to achieve a simulation as accurate as possible by porting the ISA100.11a software stack of commercial devices. This unique approach should allow the simulation to replicate the exact behavior of a physical IWSN.

**Figure 3:**
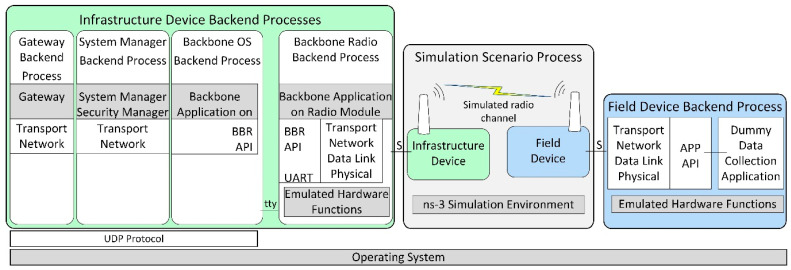
The architecture of the implemented ISA100.11a simulation model.

Figure 3 illustrates the architecture of the developed ISA100.11a simulation model, replicating the internal architecture of the physical ISA100.11a devices presented in Figure 2.

The simulated infrastructure and field device models are formed by a set of backend processes that run inside the operating system (Ubuntu 18.04 Linux distribution) instead of running on dedicated hardware.

The infrastructure device backend processes are as follows:

- Backbone Radio Backend Process, corresponding to the transceiver part of the real infrastructure device. It includes the emulated hardware functions submodule, which allows running the embedded software Backbone Application on a Radio Module without physical hardware.
- Backbone OS Backend Process, corresponding to the BBR application of the infrastructure device;
- System Manager Backend Process, corresponding to the SM application of the infrastructure device;
- Gateway Backend Process, corresponding to the Gateway application of the infrastructure device.

The Backbone OS, System Manager, and Gateway Backend Processes communicate with each other by means of the UDP protocol. A serial interface (tty) ensures communication between the Backbone OS Backend Process and the Backbone Radio Backend Process.

The Field Device Backend Process hosts several backend threads that communicate through sockets with the simulated field devices and run their functionalities, such as the ISA100.11a communication stack, the integration API, and a dummy data collection application. In the implemented basic scenarios, there is only one field device and thus only one backend thread, while in the scenarios evaluating the scalability of the simulator, there are multiple field devices and multiple backend threads.

The Simulation Scenario Process includes ns-3 and manages simulation time, simulated events, and network topology. It also implements the radio channel model, as well as simple models of the field and infrastructure devices that dynamically launch their backend processes/threads and forward simulation events to/from these backends (through Unix Domain Sockets, denoted with S in Figure 3).

During simulation startup, the Infrastructure Device and the Field Devices are created in ns-3 by instantiating ns-3 Nodes and specific ns-3 Applications. The Field Device Application is initialized, which configures the EUI64 address of its corresponding field device and loads provisioning data for it. A thread in the Field Device Backend Process is created, and communication with it is established by a Unix Domain Socket. In addition, at startup, the Infrastructure Device is initialized, and it first configures its EUI64 address and provisioning data; then, it launches its Backend Processes: (1) the Backbone Radio, (2) the Backbone OS, (3) the System Manager, and (4) the Gateway Backend Processes. Communication between the ns-3 Simulation Environment and the Backbone Radio Backend Process is established by using another Unix Domain Socket.

At the start of the simulation, an event is generated by each Field Device Application and Infrastructure Device Application. On the execution of these events, an Event Request simulation message is sent to each corresponding Field Device Backend Process thread and Backbone Radio Backend Process. In addition to their type, these simulation messages contain the current time in the simulation. When each Backend Process receives this event, it (1) updates timing-related simulated registers in its Emulated Hardware Functions module, (2) possibly executes interrupt handler routines, and (3) runs its internal main loop. While executing the program sequences from above (interrupt handlers and main loop), the ported software stack might (a) change the RF channel on which its virtual transceiver might receive RF frames, or (b) it might schedule an IEEE 802.15.4 compliant RF frame for transmission at a specific time moment—typically in less than 5 milliseconds time from the current simulation time. In the above two situations, specific simulation messages are transmitted back asynchronously to the ns-3 Simulation Environment using the Unix Domain Socket corresponding to the device. When a Backend Process’ thread finishes processing the current event, it sends an event response message to the ns-3 Simulation Environment. This response message contains the latest time-moment when the specific Backend Thread expects to be run again. It might run earlier if some event has an effect on it, for example, when a Field Device receives an incoming RF frame.

In case of RF frame transmission, an RF frame transmission complete simulation message is sent to the Backend Thread initiating the transmission, at the simulation moment when this message is generated on physical hardware. This type of message contains the success/fail status of the transmission.

RF frames transmitted by Field Devices are processed in the ns-3 Simulation Environment by the model of physical topology and RF channel. The Carrier-Sense Multiple Access medium access scheme is modeled, and in case an RF frame is received by a device, a simulation message request is sent to its corresponding Backend Process thread, containing the timestamp, RF channel, and data payload. If the virtual transceiver’s status in the Backend Process thread is in receive mode, then the RF frame is delivered to the ISA100.11a protocol stack. When the processing of the RF frame is complete, the Backend Process sends a simulation message response to the ns-3 Simulation Environment.

The Backbone Radio Backend process works similarly to a Field Device Backend Process, but it also communicates on its serial interface (tty) using BBR API messages. This interface is used to transfer data between the Backbone Radio Backend Process and other processes from the Infrastructure Device Backend Process group.

For achieving real-time functioning, the ns-3 Simulation Environment periodically waits for the wall-clock time to “catch up” with the simulated time. For this mechanism to work, the time in simulation must be at least as fast as the wall-clock time. In the current implementation, the synchronization period is 50 ms.

The simulation events include: passing of time, start/end of a radio frame transmission/reception, start/end of transmission/reception on emulated UART, and debug information transmission. The simulation scenario mirrors, as close as possible, the start-up sequence of an IWSN. As such, the devices startup, and the modules of the infrastructure device interconnect and begin the creation of the IWSN by sending advertisement radio frames. When the field devices receive the advertisements, they initiate the joining process by transmitting radio frames. The joining requests are processed by the infrastructure device, which sends responses back to the field devices. After several messages, the joining procedure is finalized, and the infrastructure device configures the field devices to start sending the collected environmental data. When this configuration is complete, the IWSN enters its steady state where most of the traffic consists of collected data and diagnostic data transmitted by the devices.

## 6. Evaluation of the ISA100.11a Model Implementation

To evaluate the accuracy of the implemented simulation model, we compare the behavior of the simulated network with the behaviors of one physical IWSN and two HIL IWSNs. To evaluate the scalability of the IWSN model, we compare metrics collected from two extended physical networks with the equivalent simulated IWSNs. The extended IWSNs contain one infrastructure device and five and ten field devices, respectively.

The *Physical network* scenario implements a real IWSN, employing a CDS VR950 gateway and a CDS VS210 node, both being commercial physical devices implementing the ISA100.11a IWSN standard. This scenario allows us to assess the performance of the real IWSN and consider the network behavior as a reference for the scenarios that employ simulation.

To validate the simulation model and make sure of its compatibility with the real ISA100.11a specifications, we make use of two HIL testing scenarios: one that tests the functionality of the infrastructure device, and another that tests the functionality of the field device. The *Gateway-in-the-loop* scenario employs a physical gateway to manage the simulated ISA100.11a network, while the *Node-in-the-loop* scenario uses a simulated gateway to manage a physical ISA100.11a network.

**Figure 4:**
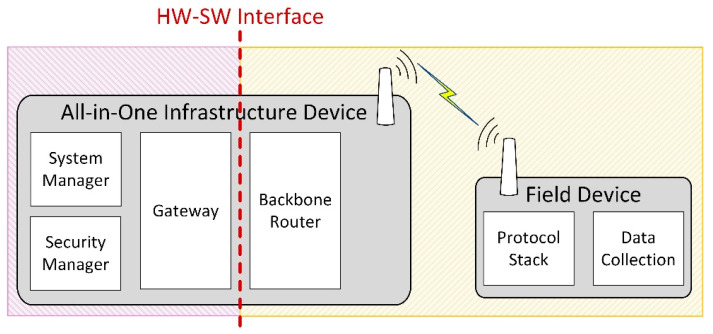
HW-SW interface in the HIL scenarios.

To avoid the potential issue of excessive delay between the hardware and software parts of the HIL system, we set the Hardware–Software (HW-SW) interface in a less time-critical part of the system. This HW-SW interface separates the physical hardware components of the testing scenario from the simulated components. In our case, this is the interface between the Backbone Router and the Gateway modules of the infrastructure device. In the HIL scenarios, the physical and simulated components can communicate only through the HW-SW interface of the infrastructure device, as illustrated in Figure 4. This way, the time-critical parts, including the Physical and Data-Link layers of the devices in the simulation, run in the same time-domain: either all of them are simulated in case of the *Gateway-in-the-loop* scenario or they use the real hardware implementation in case of the *Node-in-the-loop* scenario. As such, the testing results should showcase similar behaviors for the networks that have the same type of PHY and Data link implementations (i.e., Simulated vs. *Gateway-in-the-loop* and Physical vs. *Node-in-the-loop*).

A basic IWSN is implemented in each test scenario, consisting of one infrastructure device and one field device placed 1 m from one another. The network performance is evaluated in terms of specific network formation and operation metrics.

During a test scenario, the infrastructure and field devices are started at the same time. Next, the infrastructure device forms the IWSN, and the field device joins the network and sends data to the infrastructure device at 15-s intervals. The run-time of each test scenario is 40 min, and each test scenario has been repeated 40 times. The simulations run by wall-clock time, so that one simulated second corresponds to one real-time second.

The results of the simulations are logged in files containing the history of events from the simulator. For the hardware-based scenarios that employ a real wireless communication channel (i.e., *Physical Network* and *Node-in-the-loop*), the ISA100.11a messages transmitted over the radio interface are captured using a generic IEEE 802.15.4 sniffer that listens simultaneously to all 16 radio channels defined in the standard. The model of the sniffer is a WirelessHART Wi-Analys Network Analyzer [54].

For the simulated network, the architecture in Figure 3 is used. The hardware-based scenario architectures are described below.

### 6.1. Physical Network Scenario

**Figure 5:**
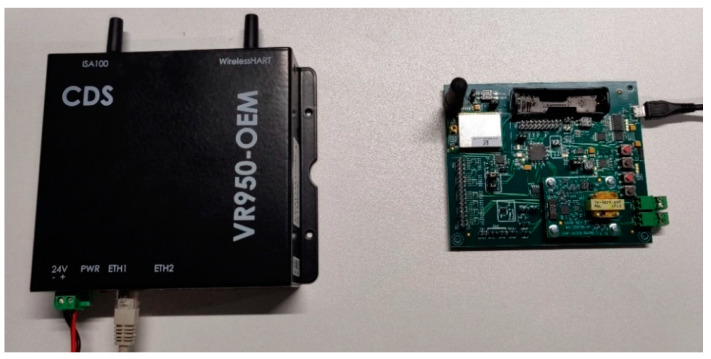
The ISA100.11a certified devices employed in the experimental setup: CDS VR950 gateway (**left**) and CDS VS210 field device (**right**).

The *Physical network* scenario implements a real IWSN, employing a CDS VR950 gateway and a CDS VS210 field device. Figure 5 shows the hardware used in the experiment. On the left side is the infrastructure device. It uses its Ethernet connector for both communication and power (Power over Ethernet technology). On the right side is the physical field device. It is powered through the USB connection. Both devices have been previously configured for the same ISA100.11a subnet ID and join key. For the VR950, these configurations have been introduced by using its web interface, while for the VS210, the Field Tool FT210 [55] has been used.

To evaluate this scenario, the log files from VR950 and the logs of the RF sniffer are used.

### 6.2. Gateway-in-the-Loop to Manage a Simulated Node

**Figure 6:**
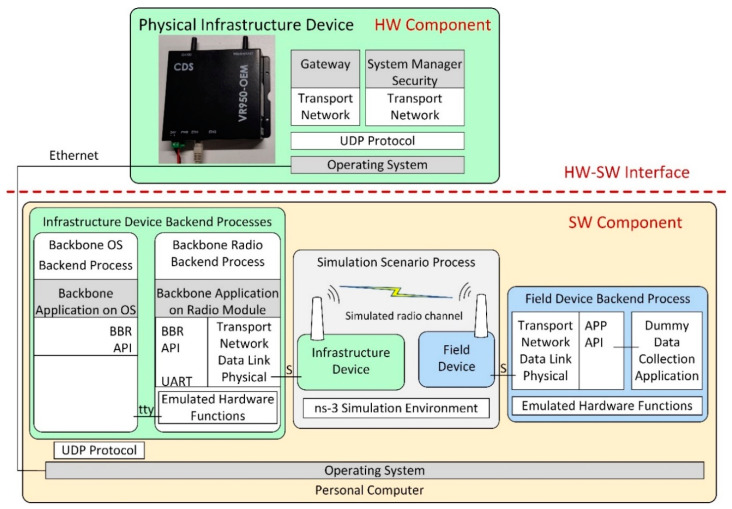
Gateway-in-the-loop scenario architecture.

This Gateway-in-the-loop scenario is presented in Figure 6. The System Manager, Security Manager, and Gateway applications are the ones implemented on the physical infrastructure device, while the Backbone modules (i.e., the Backbone OS Backend Process and Backbone Radio Backend Process) are simulated and together with the Simulation Scenario Process and Device Backend Process are running on a PC inside an operating system. The VR950 and the PC are connected through an Ethernet network, and their IP stack is properly configured for communication.

The System and Security Manager application communicates with the Backbone OS Backend Process through the UDP protocol. It configures the Backbone Radio Backend Process as it would be running on a physical gateway, and thus, the physical gateway manages the simulated network.

For this scenario, the modified physical gateway has to be started up manually before the simulation can be started on the PC. An important aspect is that the time of the VR950 and that of the PC have to be well synchronized for this scenario to succeed. Time information is used for encryption and authentication in ISA100.11a, and if the time difference between modules is greater than 60 s, then the authentication of packets fails, and the applications or processes refuse to communicate with each other. Since no physical RF transmissions take place, the necessary experimental data have been extracted from the simulation log files and the log files of the VR950 gateway.

### 6.3. Node-in-the-Loop Managed by a Physical Gateway

**Figure 7:**
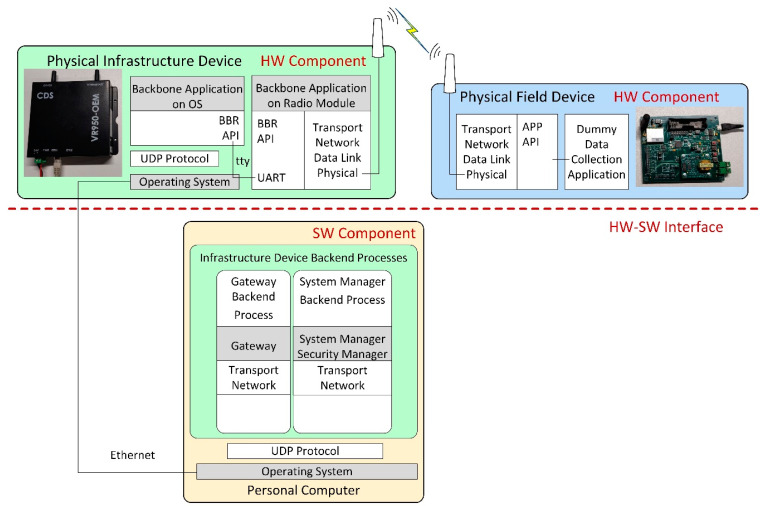
Node-in-the-loop scenario architecture.

In the *Node-in-the-loop* scenario, illustrated in Figure 7, the System and Security Manager and Gateway applications of the infrastructure device are running on a PC, while the Backbone application is running on the physical infrastructure device and communicates with the other modules through UDP protocol. The Backbone module creates a physical IWSN and allows the physical field device to communicate with the infrastructure device.

Time synchronization between the hardware and simulated parts of the scenario is important to prevent the ISA100.11a applications or backend processes from discarding received packets due to decryption or authentication errors.

The logs from the simulated System and Security Manager and Gateway and the data collected by the RF sniffer are used to evaluate this experiment.

### 6.4. Extended Real and Simulated Network Scenario

For evaluating the scalability of the proposed simulator, two extended IWSNs have been implemented both in physical form and in the simulator. These IWSNs consist of a CDS VR950 gateway, as infrastructure device, and five or ten CDS VS210 nodes connected to the gateway. The extended IWSNs are deployed in the same laboratory as the basic networks, experiencing similar environmental conditions.

**Figure 8:**
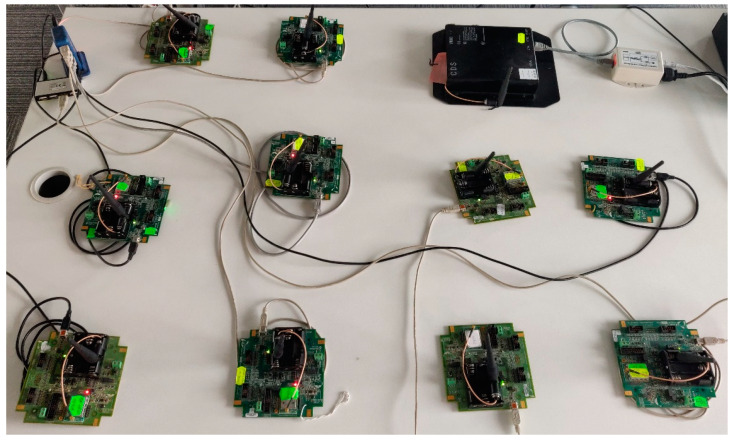
Extended real scenario implementation.

Figure 8 shows the testbed for the extended physical network. There is one infrastructure device and ten field devices. Depending on the test scenario, either five or 10 devices are powered up.

To successfully simulate IWSNs with multiple field devices, there are two key aspects that require special attention: (1) the implementation of the medium access channel model, and (2) the selection of randomness source. For (1), we modeled the RF channels such that if there is an ongoing transmission on a given RF channel, then that channel is considered busy, without taking into account the RF propagation delay between a transmission of one field device and carrier sense operation of another device. This is needed because ISA100.11a IWSNs work with synchronized timing, and at startup, each device will try to transmit at the exact same time. For (2), randomness is used in conjunction with CSMA in the exponential back-off algorithm and used for generating some cryptography-related messages. Not having random values with sufficient unpredictability can cause the IWSN to not function correctly, for example by generating too many collisions during RF medium access and practically jamming the starting-up IWSN. The pseudo-random number generator provided by the Linux OS (e.g., rand() function) is sufficiently unpredictable for making the simulated IWSN work correctly, although using it makes it harder to debug the simulator. Using a constant seed value for the pseudo-random number generator allows reproducibility of the simulation runs. Predictable and easier to debug approaches such as deriving values from current simulation time and in-simulation device identifiers by using bit-level operations make the simulated IWSN work incorrectly.

**Figure 9:**
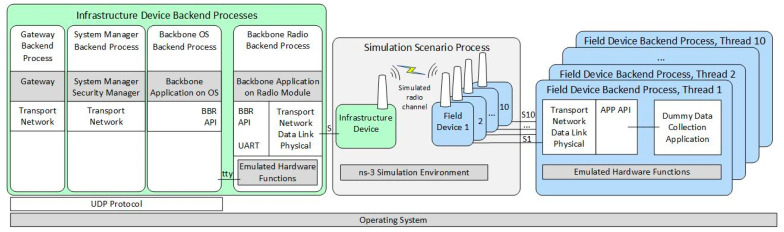
Extended simulated scenario architecture.

Figure 9 presents the architecture of the extended simulation scenario. The architecture follows the generic simulation architecture presented in Section 5.2. As illustrated in Figure 9, in the simulation scenario, there is one simulated infrastructure device and there are up to ten simulated field devices, each consisting of the field device model in ns-3 and a thread in the Field Device Backend Process. Each field device model communicates with its corresponding Field Device Backend Process by a dedicated Unix Domain Socket, which is denoted with S1 to S10. In the evaluation scenarios with five simulated field devices, there are five field device models and backend process threads, instead of 10.

These evaluation scenarios have similar behavior to the other evaluation scenarios: an IWSN is started, and it is run for a total of 40 min. All field devices have been configured to transmit data at 15-s intervals. The evaluated metrics are collected for 40 runs of each scenario. The experimental runs have been executed automatically by implementing and using controller software that starts and stops the software modules on the infrastructure device and turns on and off the field devices by controlling a programmable power switch. In case of physical IWSNs, the metrics have been extracted from the log files of the VR950 gateway and from the logs of the RF sniffer. For simulated IWSNs, the metrics have been extracted from the log files of the simulator.

## 7. Results Analysis

**Figure 10:**
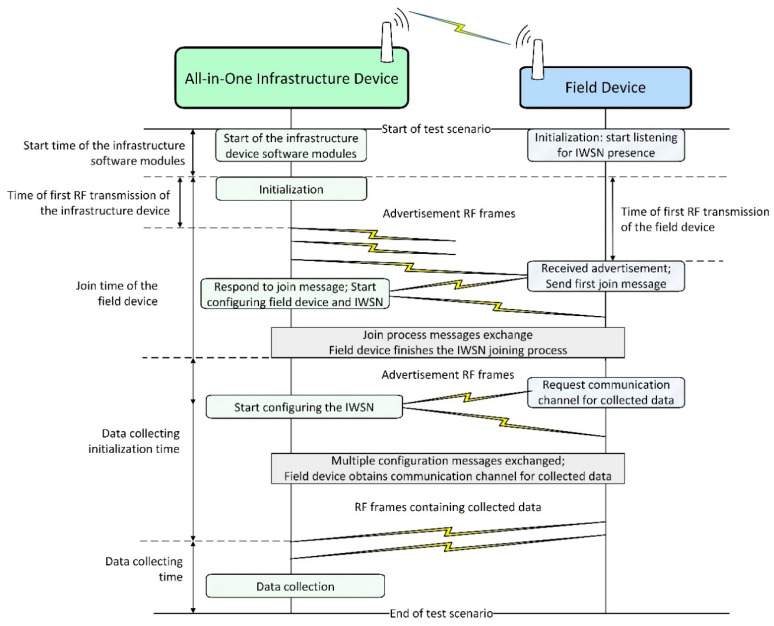
Summary of field device joining to application messages sequence in an ISA100.11a IWSN.

The evaluation of the test scenarios is performed using two sets of metrics: one related to the timing of events in the IWSN and one related to communication statistics. Several key events are taken into account when analyzing the network behavior and defining these metrics. Figure 10 illustrates these events and the message exchanges between the infrastructure device and field device during IWSN formation (including initialization, advertising, and joining processes).

As depicted in Figure 10, first, the all-in-one infrastructure device and the field device perform their initialization. Next, the infrastructure device starts broadcasting the presence of an ISA100.11a network by transmitting advertisement RF frames while the field device listens for advertisements. When the field device receives a suitable advertisement (i.e., with the network identifier and security key matching the device provisioning configuration), it starts the network joining process by sending a specific join request to the infrastructure device and then waits for a response. After the join process messages exchange, the field device is admitted in the standard-compliant network, and a management communication channel is configured between the infrastructure and field devices. Next, the field device requests a communication channel (specifically an ISA100.11a Contract) to transmit collected data and the infrastructure device allocates communication resources. When the communication channel is set up, the field device starts transmitting collected data to the infrastructure device. At this point, the field device is “joined to application” [2].

Timing information is based on the identification of specific events in the log files and storing the associated timestamp of the events. The timing information metrics are the following:

- *Start time of the infrastructure devicesoftware modules*—time elapsed between the start of the test scenario and the moment the software modules of the infrastructure device become operational.
- *Time of first RF transmission of the infrastructure device*—time elapsed since the infrastructure device becomes operational until the first RF frame is transmitted by the infrastructure device; the first RF transmission is an advertisement frame signaling the presence of the ISA100.11a IWSN.
- *Time of first RF transmission of the field device*—time elapsed since the infrastructure device becomes operational until the first RF frame is transmitted by the field device.
- *Join time of the field device*—time elapsed since the infrastructure device becomes operational until the field device is admitted in the network.
- *Data collecting initialization time*—time elapsed since the completion of the joining process until the first collected data sample is received by the infrastructure device.

**Figure 11:**
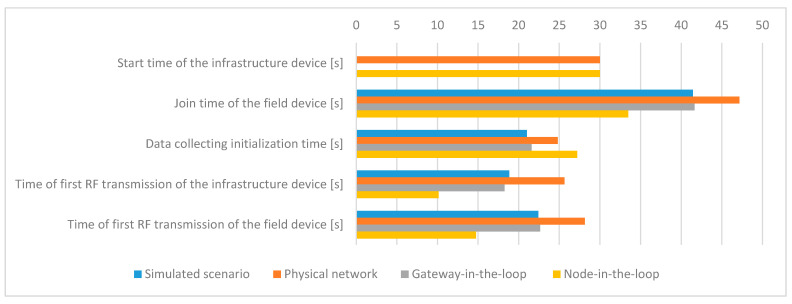
Mean values of the timing information metrics.

The mean values of the evaluated metrics related to the timing information for all scenarios are presented in Figure 11.

Communication statistics have been obtained by parsing the IEEE 802.15.4 compliant RF communication frames collected in the log files of the RF sniffer and of the simulator. The extracted communication metrics are the following:

- *Number of collected data samples received by the infrastructure device*;
- *Number of RF advertisement frames transmitted by the infrastructure device*;
- *Number of RF advertisement frames transmitted by the field device*;
- N*umber of RF communication frames transmitted by the infrastructure device*—these are management messages transmitted during the *Data collecting initialization time*;
- *Number of RF communication frames transmitted by the field device*—these are transmitted during the *Data collecting initialization time* and during *Data collection* and include the answers to the RF communication frames transmitted by the infrastructure device, collected data publishing, and device diagnostic messages;
- *Number of RF acknowledgment (ACK) frames transmitted by infrastructure device*;
- *Number of RF ACK frames transmitted by field device*.

**Figure 12:**
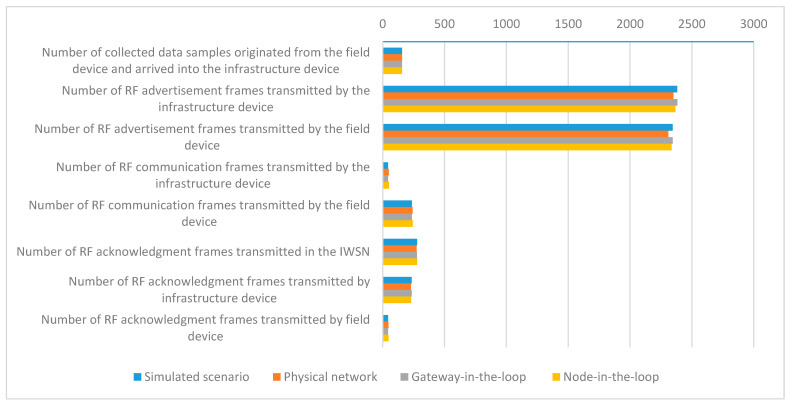
Mean values of the communication statistics metrics.

The mean values of the evaluated metrics related to communication statistics for all scenarios are presented in Figure 12.

These results show that after power-on, the physical infrastructure device requires some time to initialize its hardware, load and start the OS, and start the management modules, resulting in a 30 s delay during IWSN formation. In the simulated scenario, the OS is already running, and the start-up time of the software modules on the infrastructure device is negligible.

The tests show that more time is needed in the experimental testbed for a device to join the network once the IWSN is formed. This may occur due to retransmissions or due to CPU load on the physical infrastructure device.

*The time of first RF transmission of the infrastructure device* shows the moment when the infrastructure device becomes fully operational. The results show that this latency is the biggest in the *Physical network* scenario, being caused by the limited processing capability (CPU performance) of the physical devices. As part of the modules are simulated in the HIL scenarios, the CPU available on the computer speeds up the process, resulting in a smaller value for the *Gateway-in-the-loop* scenario and reaching the shortest time of the first RF transmission for the *Node-in-the-loop* scenario. Moreover, an inspection of the System Manager log files shows that the frequency of exchanged messages is highest for the *Node-in-the-loop* scenario.

Thus, the *Physical* scenario has the biggest value because all processing is performed on the infrastructure device, the *Gateway-in-the-loop* scenario has an intermediate value as the BBR application has been moved into the simulator, and the *Node-in-the-loop* scenario has the smallest value as the whole SM has been moved into the simulator.

Regarding the *Simulated* scenario, the log files show that there is an implementation limitation on the communication path between the Backbone Radio Backend Process and Backbone OS Backend Process (serial interface and emulated hardware functions), limiting the frequency of message exchange between the simulated transceiver and SM and by extension increasing the value of this metric. This tells us that an improved version of the proposed simulator should allow the configuration of the processing capabilities of the hardware. As such, the software modules’ initialization time would be more accurate.

The *Simulated* and *Gateway-in-the-loop* scenarios have very close values for this metric, showing that the simulated and physical network management modules behave very similarly when managing a simulated field device.

The *Time of first RF transmission of the field device* has to be correlated with the *Time of first RF transmission of the infrastructure device*. The difference between these two metrics shows the amount of time needed for the field device to find the IWSN formed by the infrastructure device. The values for these differences are similar in all scenarios. The absolute values show that the data is valid, as the field device starts the join procedure only after the infrastructure device has started signaling the presence of the IWSN.

*Data collecting initialization time*, which is defined as the time required for a device to start sending data once the IWSN is formed, has similar values in all scenarios. This metric depends on the completion of a sequence of messages; thus, it is very sensitive to delays in communication. Such delays might appear due to non-ideal RF communication conditions. Therefore, the *Physical network* and *Node-in-the-loop* scenarios are characterized by a slightly longer *Data collecting initialization time*.

The networks in the testing scenarios show comparable results for the IWSN communication metrics, the closest similarities being obtained for the networks with the same type of PHY and Data link implementations (i.e., *Simulated* vs. *Gateway-in-the-loop* and *Physical* vs. *Node-in-the-loop*).

The simulated infrastructure and field devices transmit more RF advertisement and communication frames than the physical ones because the simulated IWSN forms a few seconds earlier.

### 7.1. Accuracy of the Evaluated Metrics

For each metric and for each scenario type (i.e., Physical network, Simulated scenario, Gateway-in-the-loop, and Node-in-the-loop), we have obtained a set of results, each corresponding to the execution of a specific scenario. The values in these sets of results differ between scenario executions, so for the purpose of evaluating the simulator, we compute the mean value for each metric over multiple executions of each scenario type. There is an uncertainty attached to the mean value of each metric, because it is not known if the mean value of a finite number of scenario executions is equal to the mean value that would be obtained if the experimental scenarios would have been executed many more times.

The accuracy of the evaluated metric is defined as the closeness of the computed mean value of each metric in the simulated scenario or in the HIL experiment to the corresponding reference mean value collected in the physical deployed replica.

Moreover, we define statistical confidence intervals to quantify the precision of the computed mean value of each evaluated metric. The confidence intervals are computed from the set of the values obtained by multiple executions of a specific scenario/experiment. For each evaluated metric, its associated confidence interval is the range of values in which the computed mean value of that metric is located with the probability of the confidence level selected for the evaluation. We have selected the confidence level of 95%.

Table 4 summarizes the statistical confidence interval of the extracted metrics at a confidence level of 95%.

For the metric *Start time of the infrastructure software modules*, there is no confidence interval defined as it has no variation.

The confidence interval is presented as a percent of the mean value of its associated metric. The confidence interval for most metrics is under ±2.5%, showing that the metrics have a sufficiently precise value for our study. There are three metrics with confidence intervals greater than ±2.5%: *Data collecting initialization time*, *Time of first RF transmission of the infrastructure device*, and *Time of first RF transmission of the field device*. All these metrics are in the category of timing information, so any delay in processing or communication affects them, including retransmissions, packet loss, and interference.

Considering the order of the events from which the metrics are calculated, it can be observed that the confidence interval becomes larger with later events. This shows that randomness is added to the metrics as each test scenario is executed. Interestingly, in the case of the *Join time of the field device*, randomness seems to be decreasing, as it has a smaller confidence interval than the time of the *First RF transmission of the field device*. It is possible that this decrease in variation is related to the periodic nature of the ISA100.11a communication resources and that events tend to align with time moments when the periodic communication resources are active (i.e., ISA100.11a links, superframes, communication contracts).

### 7.2. Variations of the Evaluated Metrics

The variations of the collected metrics show how much uncertainty is associated with the metrics collected in the test scenarios. Firstly, this shows how easily repeatable the experiments are, and secondly, for the simulated scenario, it characterizes the robustness of the proposed simulator.

The variations of the evaluated metrics are quantified as the relative standard deviation of each metric, which are calculated on the 40 executions of the scenario where the metric belongs. The relative standard deviation is the statistical standard deviation of the values of its associated metric in one type of scenario, converted to a percent of the metric’s mean value. Table 5 presents an overview of the relative standard deviations of each evaluated metric.

In case of the *Start time of the infrastructure software modules* metric, there is no standard deviation defined, as it has constant values and no variation

The metrics related to timing information have generally a bigger standard deviation as a percent of their mean value than the metrics related to communication statistics. This can be explained by the fact that getting to a specific moment in the evolution of a test scenario is more sensitive to any delay than the total number of occurrences of a specific event. In the case of timing-related metrics, each random delay increases the variation of the metric.

The IWSN communication statistics indicate that both the reference scenario and the simulated and HIL scenarios show similar behaviors across multiple executions, having their relative standard deviation under ±5%.

The evaluated metrics show that the simulated IWSN behavior closely resembles the one of the IWSN deployed on the experimental testbed, demonstrating that the ISA100.11a model is accurately integrated with ns-3.

### 7.3. Scalability of the Proposed IWSN Model

The scalability of the proposed simulator has been evaluated in terms of metrics related to IWSN functionality and metrics related to computer resource usage. The metrics related to IWSN functionality are the following:

- *Data collecting initialization time for the first device to start transmitting data*, which is measured from the start of the scenario until the first collected data sample arrives to the infrastructure device. This metric is highly dependent on the behavior of medium access mechanism, as during start-up of the IWSNs RF, collisions are expected.
- *Data collecting initialization time for the last device to start transmitting data*, from the start of the scenario. This metric marks the end of the initialization of an IWSN, and it is very sensitive to the behavior of the medium access mechanism, as any failed transmission can cause significant increase in this metric.
- *Number of collected data samples received by the infrastructure device*. This metric characterizes the correct functioning and stability of the IWSN.
- *Total number of RF advertisement frames transmitted in the IWSN*. Since RF advertisement frames are transmitted periodically, their number is correlated with the level of RF interference detected by the IWSN.
- *Total number of RF communication frames transmitted in the IWSN* by the infrastructure device or by any of the field devices. This number characterizes the stability of the IWSN, as the System Manager located in the infrastructure device reacts to communication diagnostics sent by the field devices and transmits configuration messages into the network for avoiding potential issues and to optimize the network. These configuration messages are sent in RF communication frames; thus, a greater number of RF communication frames indicates that the System Manager has sent more configuration change messages to the field devices.

The relative standard deviation values of the scalability metrics are summarized in Table 6. Each scenario, simulated and physical, has been executed 40 times.

The data collecting initialization time of the simulated and physical networks is quite similar, both as mean value and as relative standard deviation of the experimental runs. The simulated networks have started data collecting some seconds earlier than the physical networks—this is probably related to the implementation of the carrier sense multiple access (CSMA) mechanism in the simulator. There might be a difference between the CSMA behavior of a physical and simulated medium, such that the simulation allows more aggressive access to the RF channel, thus allowing a simulated field device to finish the configuration message exchanges earlier and to start sending data to the infrastructure device earlier than the first device in the physical network scenario.

The fact that all five and respectively 10 field devices started collecting and sending data to the infrastructure device show the viability of the proposed simulator architecture. The mean value of data collection initialization time for the last device is much larger in the case of simulated scenarios compared to the physical networks, for both scenario types. In addition to the large difference between simulated and physical IWSNs, the relative standard deviation of the two simulated scenario types shows a large variation of this metric across multiple runs of the scenarios. Apart from the probable difference in CSMA behavior between physical and simulated scenarios mentioned above, the large variation between simulation runs shows that there is a higher amount of randomness in the simulation than in the physical network. Randomness is required for simulating an ISA100.11a communication stack, for example, in the implementation of the exponential back-off algorithm used in conjunction with CSMA; however, such large differences show that either (a) the expected distribution of the used random values is not the expected one or (b) there is some software limitation that generates this behavior.

The collected data samples in simulated and physical IWSN scenarios have similar mean and standard deviation values. The simulated scenarios have smaller values for this metric than their physical network equivalents, which is consistent with the fact that during simulations, the last device has finished data collection initialization considerably later than the last device in a physical network. These similar numbers for this metric show that after initialization, the IWSNs have been performing their task, and they have been collecting data until the end of the scenario.

The number of RF advertisement frames is very similar for the simulated and physical scenarios. Increasing the number of devices from five to 10 in the IWSN decreases a little the total number of RF advertisement frames transmitted in the IWSN, showing increased interference. The used implementation of the ISA100.11a System Manager has an upper limit on the total rate at which RF advertisement frames are transmitted by the IWSN, and it splits the maximum rate of advertisement transmission between the available field devices with routing role. So, having a slight decrease in the total number of advertisements is expected.

The total number of RF communication frames has been larger in simulated scenarios than in the physical network. This shows that in simulated scenarios, the ISA100.11a System Manager has sent more notification messages to field devices than in the physical scenarios. This observation indicates that field devices in simulated scenarios have reported less-ideal communication diagnostics than the physical field devices. The cause might be related to the implementation of CSMA or to some other software limitation.

The metrics used to evaluate the computer resource usage of the simulator are the following:

- Maximum CPU usage of the simulator while running a specific scenario.
- Average CPU usage of the simulator while running a specific scenario.

All scenarios were run on a computer equipped with an Intel Core i5-4300M CPU, having a maximum clock speed of 2.60 GHz. The CPU has been configured to use the performance mode CPU frequency governor, so variations in its clock speed have been minimized. The values of the resource usage metrics are summarized in Table 7. The CPU usage is important for this real-time simulator because the simulation needs to be at least as fast as the wall-clock, as otherwise, timing-related issues might appear in the simulation.

The results show that the simulated scenario with 10 devices has not been limited by the available CPU capacity. Probably, a scenario with more devices should work well on the same computer. It can be observed that the CPU usage increases more than linearly with the number of devices in a scenario. This might be related to the nature of the RF propagation medium, where each transmitted frame is received by every other radio transceiver in its range. Probably, the simulator can be optimized for reducing CPU usage, for example by not delivering RF frames to transceivers, which are not in receiver mode. As another direction for scaling up the size of the simulated scenarios, a more powerful computer can be used, as the one used in the evaluation is a relatively old CPU equipped in a laptop computer.

## 8. Conclusions

This paper proposes a highly accurate ISA100.11a simulation model that integrates the communication stack available on commercial ISA100.11a certified devices with the ns-3 simulator.

Using the complete implementation of the communication stack and of the network management applications allows detailed system-level modeling of the IWSN. On the one hand, the observable interactions in this model span across all layers of the communication stack, from the physical layer to the application layer. On the other hand, the model allows observing all interactions between the communication stack of the field devices, the communication stack of the infrastructure device, and the network management applications (e.g., Gateway, System Manager). This wide-ranging model is expected to closely match the behavior of a physical IWSN, helping to solve several problems related to deploying IWSNs.

The accuracy and scalability of the proposed ISA100.11a simulation model are evaluated in a number of test scenarios.

Accuracy is analyzed in four different test scenarios: (1) through simulation using the ISA100.11a model implementation integrated with ns-3, (2) on an experimental testbed employing ISA100.11a certified devices, (3) by implementing a *Gateway-in-the-loop* HIL scenario, and (4) by implementing a *Node-in-the-loop* HIL scenario.

The behavior and performances of the tested IWSNs are evaluated in terms of two sets of metrics: one related to the timing of events in the IWSN and one related to communication statistics. Moreover, the confidence interval and relative standard deviation of each metric are computed to assess the precision of the proposed IWSN model.

The results of the HIL scenarios show that from a functional point of view, the simulation model is indeed equivalent to its physical counterpart. A physical infrastructure device successfully managed a simulated field device and a physical field device successfully joined a network managed by a simulated infrastructure device. The difference between the mean values of timing-related metrics of simulated and physical IWSN is under 7 s, while the difference between HIL scenarios and physical scenarios is under 16 s. For the mean values of communication statistics, the difference between the simulated and physical IWSN for each metric is under 15%. Functional equivalence allows easy exportation of provisioning data from simulation to deployable devices. Moreover, integration testing can be first performed with the simulator, which saves time and allows greater flexibility during testing and integration.

Scalability is analyzed by implementing an extended set of simulated and real scenarios with five or 10 field devices. The performance metrics for characterizing the scalability of the ISA100.11a simulation model are related to the timing of the events, communication statistics, and computer resource usage of the simulator. For the first two types of metrics, the mean value and relative standard deviation are calculated, while for the third type, the mean and maximum values are recorded. The difference between the mean value of the number of collected data samples between simulated and physical IWSNs is under 45 data samples or 7% for an IWSN consisting of five devices, and under 512 or 54% for IWSN consisting of 10 devices. The greatest difference between mean values of metrics is in the case of *Data collecting initialization time* for the last device, having a difference of 292 s or 55.34% for an IWSN of five devices and 945 s or 73.59% for an IWSN consisting of 10 devices.

The evaluation results show that the simulated IWSN behavior closely resembles the one of the deployed networks, highlighting the fact that the proposed ISA100.11a model integrated with ns-3 accurately implements the ISA100.11a communication stack functionalities.

For future work, the authors aim to extend the research and evaluate and improve the performance of the proposed simulation model for larger IWSNs. To this extent, radio channel and network topology modeling for complex scenarios will be considered. Moreover, energy consumption models will be included to enable the proposed simulation model to provide useful information regarding the trade-off between improving the QoS of the system and reducing the overall energy consumption.

## Figures and Tables

**Table 1:** Features of IWSN simulation solutions.

IWSN Simulator Reference	Focus of Work	Applicable Standards	Communication Stack, Network Management, External Interface Implementation	Validation with Physical Devices, Mixed HW/SW (HIL) Scenario	Evaluated Metrics
[24]	Security	WirelessHART	Complete WirelessHART Stack, Network Manager	No	Effect of security attack on the success rate of collected data transmission
[25]	Effects of imperfect wireless links on industrial process	WirelessHART	Complete WirelessHART Stack, Network Manager with static resource allocation; Tennessee Eastman Process Control Challenge Problem system	No, but the process is widely studied	Process parameter variation on imperfect wireless links
[28]	Comparison of simulated IWSN protocols	ISA100.11a WirelessHART ZigBee	PHY and MAC layers, simplistic application layer	No	Communication statistics, energy consumption, RF signal level
[29]	Optimization of WSN energy consumption	ISA100.11a, WirelessHART	PHY, MAC, routing layers	Model based on physical devices	Network lifetime based on energy consumption
[32]	Mathematical model for energy consumption	IEEE 802.15.4	Not applicable	Model based on physical devices	Energy consumption
[33]	Effects of processing load on communication	IEEE 802.15.4	IEEE 802.15.4 stack and PAN coordinator	No	Application performance as a function of network delay and CPU load
[36]	Discussion on IWSN deployment, focus on signal quality	ISA100.11a, WirelessHART	Not applicable	Model based on a physical network	RF signal quality
[37]	Simulation of complete WirelessHART network	WirelessHART	Complete WirelessHART Stack, Network Manager	Model validated by comparison with a physical network	Management overhead, communication statistics, reliability, energy consumption
[38]	Hardware-in-the-loop testbed	IEEE 802.15.4	TinyOS stack and network management	Validated using HIL	Energy consumption
[41]	Integrating simulation with real HW at RF level	IEEE 802.15.4	PHY, MAC layers, and application model	Validated using HIL	Signal strength
The proposed ISA100.11a simulation model	Accurate implementation of ISA100.11a communication stack functionalities	ISA100.11a	Complete ISA100.11a Stack, System Manager; external interface available in Gateway module	Validation by comparison with the identical physical network, and two HIL scenarios	Start-up timings, communication statistics

**Table 2:** Criteria used for selecting the IWSN simulator.

IWSN Simulator	License	Actively Maintained	General-Purpose Simulator	Programming Language
OMNeT++ [14]	Commercial/ Non-commercial	Yes	Yes	C++
QualNet simulator [20]	Commercial	Yes	Yes	C/C++
TOSSIM [21]	Open-source, BSD [44]	Yes	No, TinyOS specific	nesC
ns-2 [34]	Open-source, GNU GPLv2	No	Yes	C++, TCL
ns-3 [11]	Open-source, GNU GPLv2	Yes	Yes	C++, Python
Avrora [22]	Open-source, BSD [45]	No	No, WSN specific	Java
Cooja [23]	Open-source, BSD [46]	Yes [47]	No, WSN specific	Java
RTSim [35]	Open-source, GNU GPLv2	No [48]	Yes	C++
Riverbed Modeler [19]	Commercial/Academic	Yes	Yes	C/C++

**Table 3:** Protocol layers of ISA100.11a communications stack.

**ISA100.11a Protocol Layers**	**OSI Model**
User Application Processes (UAPs)	Application
Application sub-layer (ASL)/ISA native and legacy protocols (tunneling)	
Transport Layer (TL)/UDP (IETF RFC 768)	Transport
Network Layer (NL)/6LoWPAN (IETF RFC 4944)	Network
Upper data link layer/ISA100.11a upper Data Link Layer IEEE 802.15.4	
MAC extension	Data link
Media Access Control (MAC) sub-layer: IEEE 802.15.4 MAC	
Physical layer: IEEE 802.15.4 PHY (2.4 GHz)	Physical

**Table 4:** The confidence intervals of the evaluated metrics.

Metric	Simulated Scenario	Physical Network	Gateway-in-the-Loop	Node-in-the-Loop
IWSN timing information				
Start time of the infrastructure software modules	0.0 s	30.0 s	0.0 s	30.0 s
Time of first RF transmission of the infrastructure device	18.8 ± 0.75% s	25.6 ± 1.13% s	18.3 ± 6.73% s	10.1 ± 1.75% s
Time of first RF transmission of the field device	22.4 ± 2.36% s	28.1 ± 2.07% s	22.7 ± 4.48% s	14.8 ± 3.92% s
Join time of the field device	41.4 ± 1.29% s	47.2 ± 1.34% s	41.6 ± 2.43% s	33.5 ± 1.89% s
Data collecting initialization time	21.0 ± 6.52% s	24.8 ± 5.10% s	21.6 ± 6.09% s	27.2 ± 5.17% s
IWSN communication statistics				
Number of collected data samples received by the infrastructure device	156.4 ± 0.10%	156.0 ± 0.05%	156.2 ± 0.09%	156.5 ± 0.10%
Number of RF advertisement frames transmitted by the infrastructure device	2382.2 ± 0.01%	2349.9 ± 0.58%	2382.3 ± 0.05%	2367.4 ± 0.35%
Number of RF advertisement frames transmitted by the field device	2345.1 ± 0.02%	2309.5 ± 0.77%	2344.6 ± 0.06%	2335.2 ± 0.17%
Number of RF communication frames transmitted by the infrastructure device	40.4 ± 0.55%	49.7 ± 1.27%	40.3 ± 0.55%	50.7 ± 1.06%
Number of RF communication frames transmitted by the field device	236.5 ± 0.08%	241.2 ± 0.30%	236.3 ± 0.08%	243.1 ± 0.25%
Number of RF ACK frames transmitted by infrastructure device	234.5 ± 0.08%	228.1 ± 0.77%	234.3 ± 0.08%	230.2 ± 1.41%
Number of RF ACK frames transmitted by field device	43.4 ± 0.51%	46.5 ± 2.28%	43.3 ± 0.51%	47.5 ± 2.73%

**Table 5:** Relative standard deviations of the evaluated metrics.

Metric	Simulated Scenario	Physical Network	Gateway-in-the-Loop	Node-in-the-Loop
IWSN timing information				
Start time of the infrastructure software modules	0.0 s	30.0 s	0.0 s	30.0 s
Time of first RF transmission of the infrastructure device	18.8 ± 2.42% s	25.6 ± 3.65% s	18.3 ± 21.71% s	10.1 ± 5.64% s
Time of first RF transmission of the field device	22.4 ± 7.63% s	28.1 ± 6.67% s	22.7 ± 14.47% s	14.8 ± 12.64% s
Join time of the field device	41.4 ± 4.16% s	47.2 ± 4.31% s	41.6 ± 7.83% s	33.5 ± 6.11% s
Data collecting initialization time	21.0 ± 21.03% s	24.8 ± 16.46% s	21.6 ± 19.64% s	27.2 ± 16.69% s
IWSN communication statistics				
Number of collected data samples received by the infrastructure device	156.4 ± 0.31%	156.0 ± 0.18%	156.2 ± 0.30%	156.5 ± 0.32%
Number of RF advertisement frames transmitted by the infrastructure device	2382.2 ± 0.02%	2349.9 ± 1.87%	2382.3 ± 0.17%	2367.4 ± 1.12%
Number of RF advertisement frames transmitted by the field device	2345.1 ± 0.06%	2309.5 ± 2.49%	2344.6 ± 0.19%	2335.2 ± 0.54%
Number of RF communication frames transmitted by the infrastructure device	40.4 ± 1.76%	49.7 ± 4.09%	40.3 ± 1.78%	50.7 ± 3.42%
Number of RF communication frames transmitted by the field device	236.5 ± 0.27%	241.2 ± 0.95%	236.3 ± 0.25%	243.1 ± 0.82%
Number of RF ACK frames transmitted by infrastructure device	234.5 ± 0.27%	228.1 ± 2.47%	234.3 ± 0.26%	230.2 ± 4.53%
Number of RF ACK frames transmitted by field device	43.4 ± 1.64%	46.50 ± 7.35%	43.3 ± 0.00%	47.5 ± 8.81%

**Table 6:** Relative standard deviations of scalability metrics related to IWSN functionality.

Metric	Simulated Scenario, 5 Devices	Physical Network, 5 Devices	Simulated Scenario, 10 Devices	Physical Network, 10 Devices
Data collecting initialization time for the first device	57.0 ± 10.27% s	66.7 ± 10.27% s	57.8 ± 9.44% s	70.3 ± 9.47 s
Data collecting initialization time for the last device	527.1 ± 31.95% s	235.4 ± 12.94 s	1284.1 ± 40.19% s	339.1 ± 6.49% s
Number of collected data samples received by the infrastructure device	704.3 ± 2.33%	748.8 ± 0.71%	949.1 ± 6.53%	1460.6 ± 0.85%
Total number of RF advertisement frames transmitted in the IWSN	9045.2 ± 1.46%	9055.8 ± 1.30%	8653.4 ± 3.22%	8502.3 ± 2.93%
Total number of RF communication frames transmitted in the IWSN	7309.9 ± 11.62%	5248.2 ± 9.37%	16436.2 ± 10.57%	11839.8 ± 8.97%

**Table 7:** Values of scalability metrics related to computer resource usage.

Metric	Simulated Scenario, 1 Device	Simulated Scenario, 5 Devices	Simulated Scenario, 10 Devices
Average CPU usage of the simulator	4%	10%	21%
Maximum CPU usage of the simulator	5%	13%	24%
